# A rare case of human infection with Orf virus in China, 2024

**DOI:** 10.3389/fcimb.2025.1710971

**Published:** 2026-01-07

**Authors:** Miao Lu, Yumin Liang, Haijian Zhou, Hai Jiang, Xueyun Wang, Wenguo Jiang, Xihong Sun, Shuguang Zhong, Kun Li

**Affiliations:** 1 National Institute for Communicable Disease Control and Prevention, Chinese Center for Disease Control and Prevention, Beijing, China; 2 Communicable disease prevention and control, Jining Center for Disease Control and Prevention, Jining, Shandong, China; 3 Infectious disease, Jining Public Health Medical Center, Jining, Shandong, China

**Keywords:** China, genetic analysis, Orf virus, sheep, zoonotic pathogen

## Abstract

Orf virus is a globally distributed zoonotic pathogen that mainly infects sheep and goats, although cases of human infection have been occasionally reported. In this study, we reported a rare case of human infection with Orf virus in China. A 66-year-old shepherd in Jining City of Shandong Province, China, presented to the hospital with multiple purulent nodules on his right arm, left wrist, and jaw that had developed over the last 8 days. About 20 days before admission, he had come into direct contact with infected sheep. Infection with the Orf virus was diagnosed based on amplification and analysis of the viral B2L gene, which exhibited the highest nucleotide identity (99.46%) to an Orf virus strain from Russia but was relatively distant from other Orf strains from China. The F1L gene was also recovered, although it had lower identity (98.71%) to the China strains. Our results imply that clinicians in China need to stay alert for Orf infection in humans to ensure accurate and prompt diagnosis, especially in endemic areas.

## Introduction

The Orf virus (ORFV), a dsDNA virus belonging to the genus Parapoxvirus of the family Poxviridae, is the etiologic agent of contagious ecthyma in sheep and goats (Chen et al., 2017). It is distributed worldwide and is ubiquitous in almost all sheep- and goat- raising countries. In spite of its considerable economic impact in the animal husbandry industry, contagious ecthyma is a self-limiting infection that mainly manifests as lesions on the lips, tongue, and around the nostrils of infected animals (Chi et al., 2013). The symptoms usually resolve within 1 month in adult animals, but it could be fatal in kids (Ma et al., 2022).

As a zoonotic pathogen, the Orf virus can easily be transmitted to humans through contact with infected animals or contaminated animal products (Mungmunpuntipantip and Wiwanitkit, 2024). The clinical manifestations of human infection vary from small papules to severe pustular dermatitis lesions (Santiago et al., 2019). While human-to-human transmission is very rare, there have been reports of human infection (Turk et al., 2014; Rajkomar et al., 2016). The genome of Orf virus is approximately 138 kb long and encodes 132 genes. Molecular diagnosis of Orf virus infections is mainly based on the amplification and analysis of the envelope gene (B2L) (Gelaye et al., 2016), which encodes a highly conserved region of the immunogenic protein. In addition, the F1L and Vir genes are also used for phylogenetic analysis of the Orf virus and provide detailed insights into its genetic diversity and evolutionary patterns.

In China, Orf virus infection has been reported in goats and sheep from many provinces including Fujian, Shaanxi, Yunnan, and Heilongjiang (Chi et al., 2015; Yu et al., 2017). However, cases of human infection have been rare and were only occasionally reported in recent years (Liu et al., 2023; Yu et al., 2023). In 2024, we identified a case of human Orf infection in a 66-year-old man in China who presented with nodules on his arm, wrist, and jaw. He had come into direct contact with sick sheep and was diagnosed with Orf virus infection based on molecular analysis.

## Case presentation

**Figure 1:**
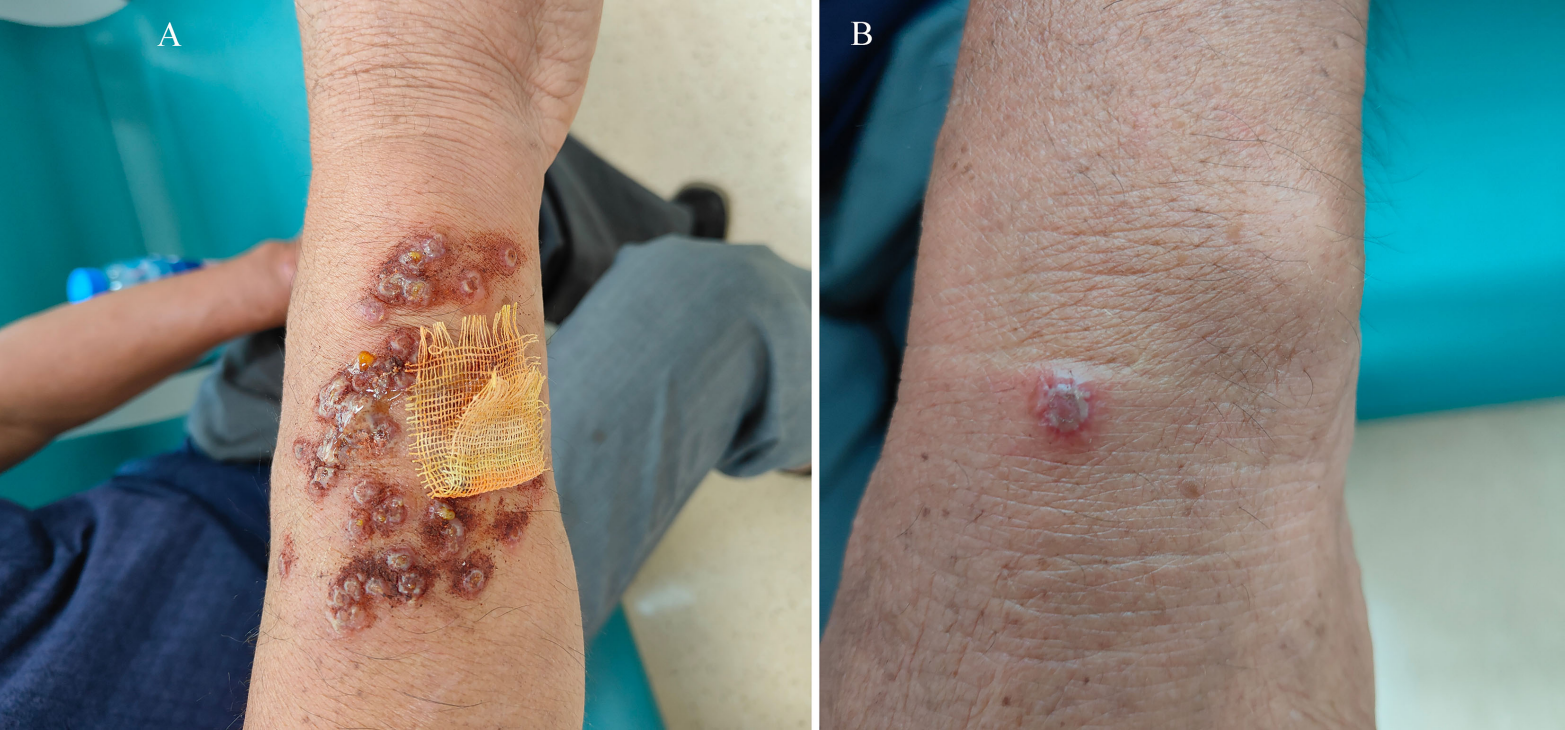
Images depicting the presentation of the infection. The images depict purulent nodules on the right arm of the patient **(A)** and a nodule on the left wrist of the patient **(B)**.

In August 2024, a 66-year-old shepherd in Jining City of Shandong Province, China, presented to the hospital with multiple purulent nodules on his right arm, left wrist, and jaw (**Figures 1A, B**) that had developed over the last 8 days. The nodules were painless and some yellow watery discharge (**Figure 1A**) was observed. He was in good general health and had no symptoms, such as fever, headache, diarrhea, fatigue, and anorexia. The results of all other routine tests were normal. He was suspected of having Mpox or Herpesvirus infection, but the laboratory test results for both pathogens were negative. Oral acyclovir and betamethasone were administered for a week, along with external application of berberine powder and Pevisone cream. However, no significant improvement was observed in the symptoms.

**Figure 2:**
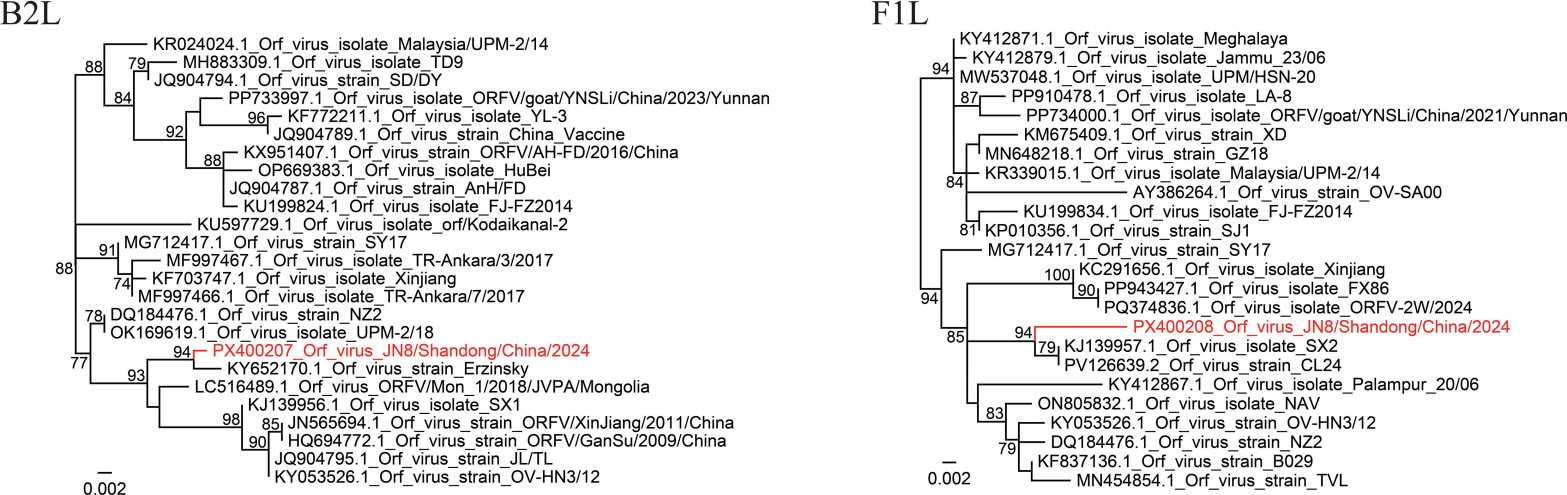
Phylogenetic tree based on B2L and F1L sequences of the Orf virus strains. The phylogenetic trees were constructed using PhyML3.0 based on the maximum likelihood (ML) method in the GTR model, and edited using FigTree v1.4.3. The confidence values for each branch were tested by bootstrapping with 100 repetitions. The Orf virus strains that were mostly closely related to the current strain, and most representative strains in China were chosen as reference sequences.

Considering the patient’s exposure to sheep, Orf virus infection was suspected. To determine whether this was the case, a pustular fluid sample was collected in an Eppendorf tube that was stored in dry ice and transported for testing to the laboratory of National Institute for Communicable Disease Control and Prevention. The DNA was extracted from the sample using a QIAamp DNA Blood Mini kit (Qiagen, Germany) and tested for the presence of Orf virus by nested-PCR using primers as shown in a previous study (Abrahão et al., 2009). The PCR amplification was performed in a PCR System LabCycler (Sensoquest, Germany). After electrophoresis, a unique and specific band (approximately 600 bp) was observed and the PCR product was then subjected to DNA sequencing. BLASTN showed that the sequence had the highest nucleotide identity (99.46%) to the Orf virus strain Erzinsky (KY652170.1) from sheep in Tuva Republic of Russia, 98.75% identity to Orf virus ORFV/Mon 1/2018/JVPA/Mongolia (LC516489.1) from goat in Mongolia, and 98.39% or lower identities to Orf virus strains from elsewhere. In the phylogenetic tree constructed using PhyML 3.0 (Guindon and Gascuel, 2003), this strain (Orf virus JN8/Shandong/China/2024) was closely related to the Orf virus strain Erzinsky and the two strains formed a relatively independent clade. To further confirm the identity of the causative pathogen, the partial F1L gene (also named 059 gene) with a length of 621 bp was also amplified and sequenced using a set of nested primers (Abdullah et al., 2015). BLASTN shows that the sequence had the highest similarity (98.71%) with Orf virus strain CL24 (PV126639.2) in sheep from Jilin Province, China. In the phylogenetic tree (**Figure 2**), this strain also represents a relatively independent branch. Additionally, the partial sequence of the virus interferon resistance (Vir) gene (436 bp) was also recovered. Interestingly, it showed 99.08% identity to Orf virus strains from Henan Province of Central China (KY053526.1) and Xinjiang Province of Northwest China (JN565695.1) (**Supplementary Figure S1**).

Subsequently, an epidemiological investigation was carried out to determine the origin of infection. The patient had been tending to a flock of 24 sheep. About 20 days before admission to the hospital, most of the sheep (about 20 sheep) exhibited festering and ecthyma around their mouths. He had come into direct contact with the affected areas while treating the sheep. The patient reported that he had not come into contact with other sheep or suspicious animals before onset. At the time the patient presented to us, the sheep he had been tending to had recovered. We did not detect Orf virus in the nasal swabs of the sheep (six samples) and the environment samples (four samples containing a mixture of stool and mud) of the sheepfold by nested PCR. Despite this, it can be inferred that the patient was infected by the sick sheep. Furthermore, the patient claimed that the flock was derived from sheep bought from a trading market in a neighboring county more than ten years ago. They were bred in captivity and had not come into contact other animals recently. Based on this information, we deduced that some of the sheep might have developed a persistent Orf virus infection.

After the molecular diagnosis of Orf infection, acyclovir and betemethasone were discontinued and replaced with cefquinome sulfate and florfenicol to prevent secondary bacterial infection. In addition, herbal cream was used as adjuvant therapy. Within a month, the purulent nodules disappeared and his skin gradually returned to normal.

## Discussion

The Orf virus is a globally distributed zoonotic pathogen that has been reported in almost all continents. In this report, we describe a rare case of human infection in Shandong Province of China. Based on our observations, we speculate that Orf infection in humans may be widely distributed in China and may, especially, pose an occupational hazard among livestock workers. Importantly, on account of the relatively mild symptoms, there is a risk of overlooking or misdiagnosing such cases.

Surprisingly, the DNA sequence of the B2L gene in the sample isolated from the present patient was highly homologous to an Orf virus strain (99.46% nucleotide identity) reported in 2015 in the Tuva Republic of Russia which is more than 2000 kilometers from Jining City. In contrast, the sequence is relatively different from any other Orf strains in China. Although the Vir gene sequence was highly homologous to strains from Henan and Xinjiang, China, the B2L and F1L genes were distant from the Henan strain (**Figure 1**). Based on these genetic and epidemiological investigations, it is difficult to determine the origin of this strain, but we suppose there might be two possibilities. 1) The current strain may be an unreported strain in domestic animals in China. 2) This strain may have been imported from other countries (Russia or the neighboring Mongolia) through livestock trading in early years. However, there is no convincing evidence of the origin, which requires further investigation in the future.

It has been reported that Orf infection could be persistent with clinically normal presentation in animals (Nettleton et al., 1996). This might make it difficult to detect all asymptomatic infected animals due to the low virus load. Therefore, more attention should be paid to control this risk. In addition, if the patients with similar symptoms have the contact history with animals, the possibility of Orf virus infection should be considered by clinicians.

This case report also has some limitations that need to be mentioned. First, the Orf virus strain was not isolated, and its genome was not sequenced. As a result, the conjecture on its possible origin was only based on partial DNA sequences of several known genes of the virus. Second, we did not detect Orf virus in the sheep or environmental samples, so the evidence chain was not complete. Third, the country or region of origin of the sheep was also not clear. In conclusion, we did not obtain convincing evidence on the molecular and epidemiological characteristics of the virus or its origin. Therefore, further investigations are needed to provide a more detailed and complete picture of the epidemiological patterns of human Orf virus infection in China.

## Data Availability

The datasets presented in this study can be found in online repositories. The names of the repository/repositories and accession number(s) can be found below: https://www.ncbi.nlm.nih.gov/genbank/, PX400207 https://www.ncbi.nlm.nih.gov/genbank/, PX400208.

## Supplementary material

Supplementary Figure S1 Phylogenetic tree based on virus interferon resistance (Vir) gene sequence of the Orf virus strains.
