# Supplementary figures and images for: A rare case of human infection with Orf virus in China, 2024
Source: Front Cell Infect Microbiol. 2026 Jan 7;15:1710971. doi: 10.3389/fcimb.2025.1710971 (PMC12819755; doi:10.3389/fcimb.2025.1710971)

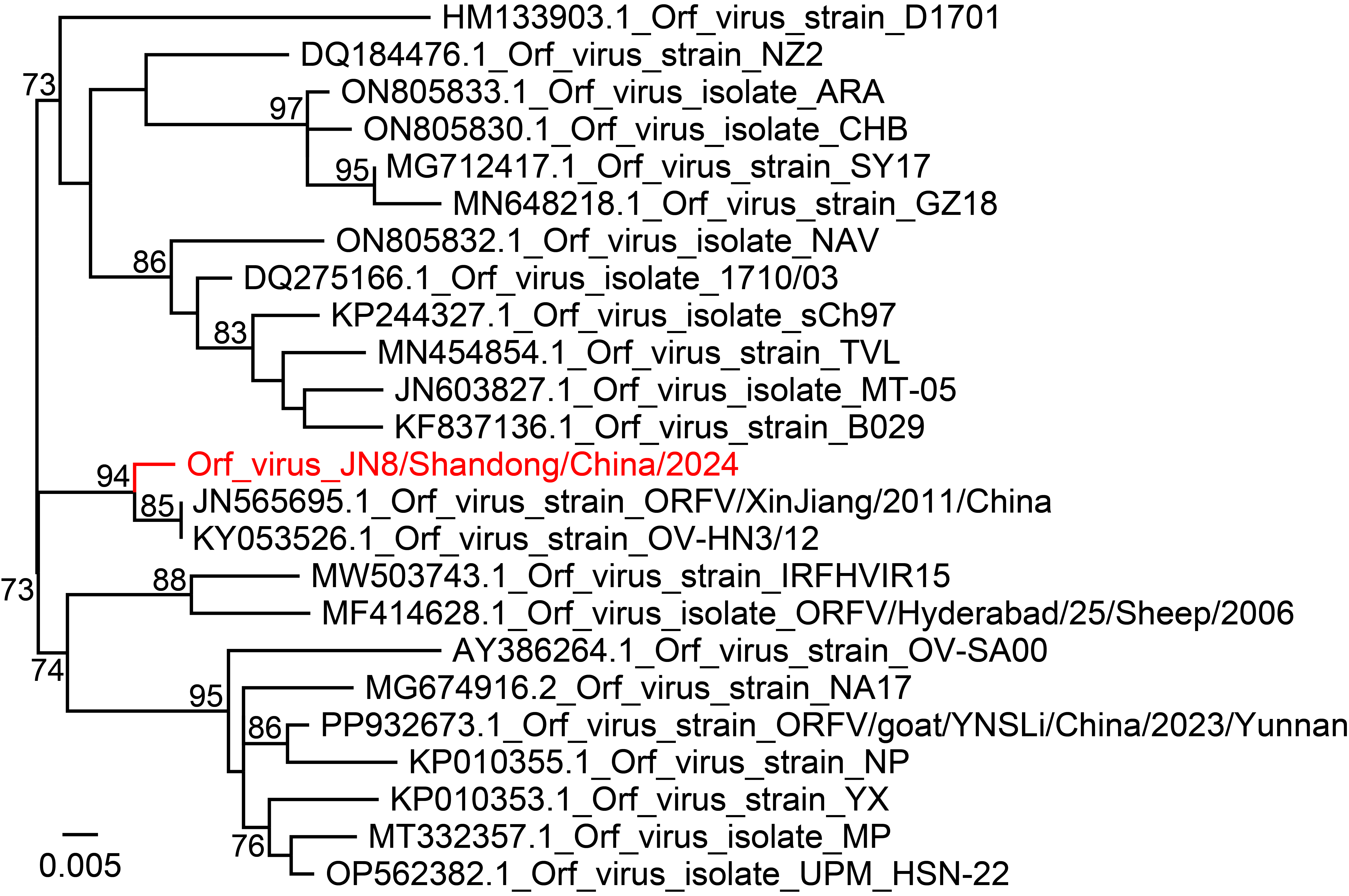

Supplement: Supplementary Figure S1 — Phylogenetic tree based on virus interferon resistance (Vir) gene sequence of the Orf virus strains. [file Image1.jpeg]
